# Improved NO_2_ Gas Sensing Properties of Graphene Oxide Reduced by Two-beam-laser Interference

**DOI:** 10.1038/s41598-018-23091-1

**Published:** 2018-03-20

**Authors:** Li Guo, Ya-Wei Hao, Pei-Long Li, Jiang-Feng Song, Rui-Zhu Yang, Xiu-Yan Fu, Sheng-Yi Xie, Jing Zhao, Yong-Lai Zhang

**Affiliations:** 1 Institute of Materials, China Academy of Engineering Physics, Mianyang, Sichuan 621900 People’s Republic of China; 2 State Key Laboratory on Integrated Optoelectronics, College of Electronic Science and Engineering, Jilin University 2699 Qianjin Street, Changchun, 130012 People’s Republic of China; 3 Center for High Pressure Science and Technology Advanced Research, Changchun, 130012 People’s Republic of China; 4 College of Electrical and Electronic Engineering, Changchun University of Technology, Changchun, 130012 People’s Republic of China

## Abstract

We report on the fabrication of a NO_2_ gas sensor from room-temperature reduction of graphene oxide(GO) via two-beam-laser interference (TBLI). The method of TBLI gives the distribution of periodic dissociation energies for oxygen functional groups, which are capable to reduce the graphene oxide to hierarchical graphene nanostructures, which holds great promise for gaseous molecular adsorption. The fabricated reduced graphene oxide(RGO) sensor enhanced sensing response in NO_2_ and accelerated response/recovery rates. It is seen that, for 20 ppm NO_2_, the response (R_a_/R_g_) of the sensor based on RGO hierarchical nanostructures is 1.27, which is higher than that of GO (1.06) and thermal reduced RGO (1.04). The response time and recovery time of the sensor based on laser reduced RGO are 10 s and 7 s, which are much shorter than those of GO (34 s and 45 s), indicating that the sensing performances for NO_2_ sensor at room temperature have been enhanced by introduction of nanostructures. This mask-free and large-area approach to the production of hierarchical graphene micro-nanostructures, could lead to the implementation of future graphene-based sensors.

## Introduction

Nitrogen dioxide (NO_2_), mainly released by automotive emissions and combustion of conventional fossil fuels, is one of the common air pollutants and can also threaten the health of human beings, because it could bring several serious diseases even at low concentrations. Therefore, the development of high performance gas sensor to detect the gas of NO_2_ in an economic way is crucially important, for not only health protection but also environmental applications. Since the ability to detect individual gas molecules by graphene was demonstrated^1^, considerable numbers of research activities have been ignited for graphene-based gas sensors^2–7^, including flexible gas sensors for wearable sensing applications^8,9^, and NO_2_ detecting sensors^10–13^, because graphene and its derivatives exhibit great electronic conductivity, flexibility, low noise and good thermal stability, it has been generally considered as a promising gas-sensing material for various highly sensitive detections. But interaction between intrinsic graphene and gas molecules is weak, in contrast, the surface of graphene oxide contains a large number of chemically active defects, showing remarkably improvement in adsorption capacity and large-scale preparation^14–18^. However, the surface area is reduced due to the sheet stacking, which seriously restricts the development of graphene-based gas sensors. In order to make the interior sheet fully contact to the test gas, it is necessary to prepared micro-nano hierarchical structure on the film to expand the specific surface area of material, and then, to improve the performance of the device. Researchers have developed nanostructured materials such as a 3-dimensional (3D) graphene foam network and a graphene nanomesh to overcome low-level sensitivity and slow response of graphene-based gas sensors^19–23^. Han *et al*. reported porous grapheme oxide network for chemical sensing via steam etching^20^. Yavari *et al*. using the method of CVD fabricated 3-D grapheme foam network for gas detection^21^. Paul *et al*. fabricatied graphene nanomesh using reactive-ion-etching for NO_2_ and NH_3_ sensing^22^. Yun *et al*. presented 3D nanostructured RGO scaffold using method of electrostatic self-assembly^23^. The method of freeze drying was also reported for the fabricating of 3D graphene/SnO_2_ structure^24^ and 3D SnO_2_/RGO structure^25^. Lupan *et al*. reported a low-powered sensor based on a microtube network^26^. These methods are limited by restricted temperature, mask, requirement of special equipment and substrate transfer or difficulty in mass production. Here we propose a strategy on the regulation of surface characteristics of graphene oxide using laser micro-nanofabrication technology, to build a high specific surface area of micro-nanostructures and fabricate graphene-based NO_2_ sensor. Because GO has good solution process compatibility, the device can be implemented on any substrate, this method does not require a substrate transfer process. Laser micro-nanofabrication can be carried out in the air at room temperature, and it has the advantage of rapid preparation of large areas. By Two-beam-laser Interference method, surface area of the film is increased, the sensitivity of the device towards NO_2_ has been enhanced, meanwhile, the response and recovery time is reduced to varying degrees.

## Results and Discussion

**Figure 1:**
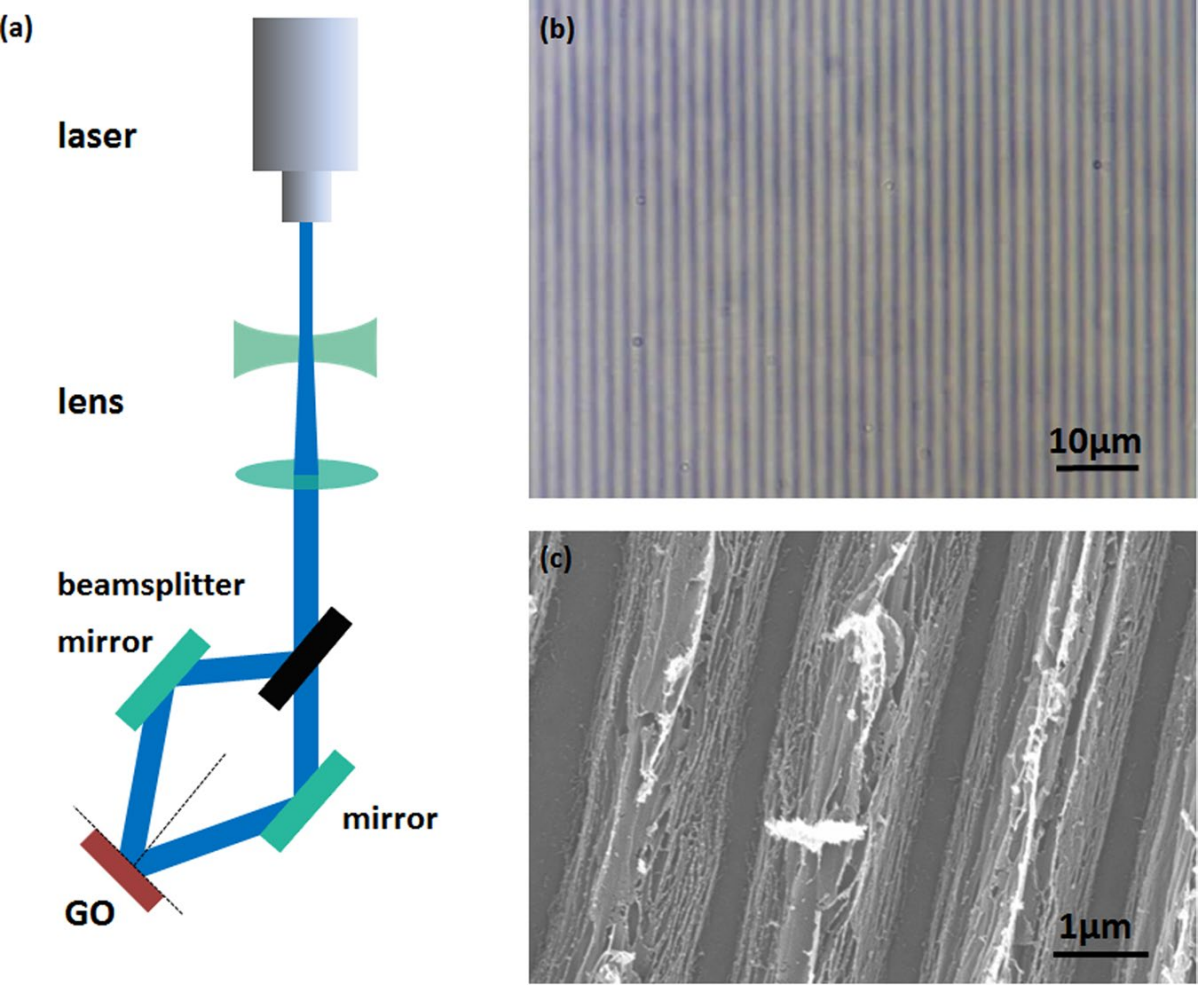
(**a**) Fabrication scheme of reduction of GO film by two beam laser interference. (**b**) Optical microscope image of RGO film. (**c**) SEM image of the grating structure of the graphene surface fabricated at 0.15 W.

In this work, TBLI was adopted for larger-area reduction and patterning of GO film towards a RGO gas sensing device. As shown in Fig. 1a, a laser beam of 355 nm wavelength was split into two branches and guided to interfere directly on the surface of a GO film. After 10 seconds of laser exposure, interference occurs. Since distribution of the laser intensity is sinusoidal^12,27^, the GO film was proposed to be reduced with a similar distribution. Figure 1b shows optical microscopy images of GO film, The periodic patterns could be clearly identified due to the difference in transparencies. SEM images of GO film was shown in Fig. 1c, GO film was patterned into hierarchical nanostructures. This layered nanostructure may result from periodic reduction and ablation of layered graphene oxide stack. The ultrafast removal of interlayer water and oxygen-containing gaseous species occurred during nanosecond laser irradiation. This layered nanostructure hold great promise for guest molecules adsorption and desorption.

**Figure 2:**
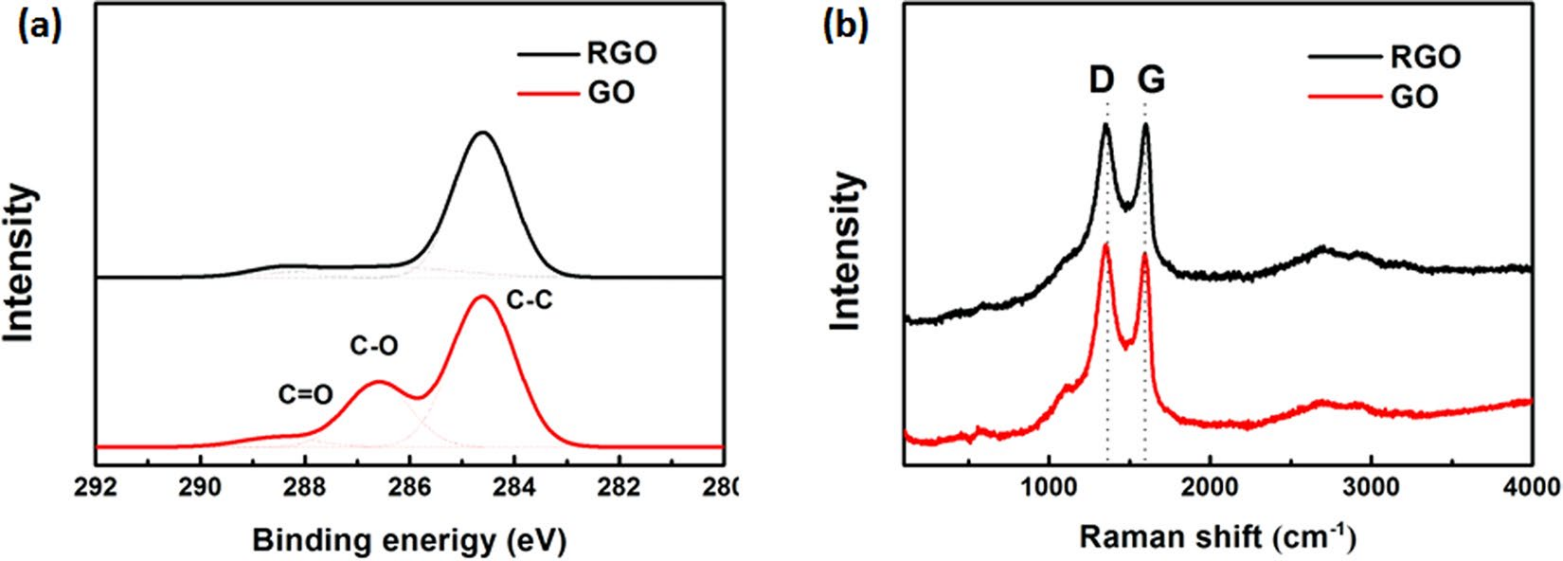
(**a**) C1s XPS and (**b**) Raman spectra of pristine GO and RGO.

C1s XPS was used to measure the surface oxygen contents. As shown in Fig. 2a, the three peaks at 284.6, 286.6 and 288.5 eV are attributed to C-C (nonoxygenated ring carbon), C-O (hydroxyl and epoxy carbon), and C=O (carbonyl), respectively. Notably, the contents of oxygen atoms in pristine GO is as high as 34.8%, the carbon not bonded to oxygen is 65%. After reduction, C-C percentage increases to 76%, indicating the successful removal of oxygen groups.

Raman spectra of pristine GO and RGO films were measured to evaluate the structural change before and after nanosecond laser reduction. As shown in Fig. 2b the I_D_/I_G_ ratios of GO and RGO were 1.07 and 0.99, respectively. A decrease of the I_D_/I_G_ ratio is perhaps an indication of graphitization.

To compare the sensing performance of GO and RGO hierarchical nanostructures, NO_2_ sensors were fabricated on ceramic substrates with interdigital electrodes with the laser power of 0.15 W, shown in Figure S1(a). Figure S1(b) presents the current–voltage characteristics of the sensor structure based on GO and RGO. The structure shows a linear behavior for both negative and positive bias voltages in between the −2 V to +2 V region, and indicates the formation of ohmic contacts between the electrode and GO(RGO). However, at higher applied bias voltages an increase in current (decrease in resistance) can be observed. This effect can be related to the self-heating effect of the aerographite-based sensing material, as was already observed for other carbon based structures^26^. After laser reduction, the electrical resistance of the device decreased from 50.2 to 11.7 kΩ.

**Figure 3:**
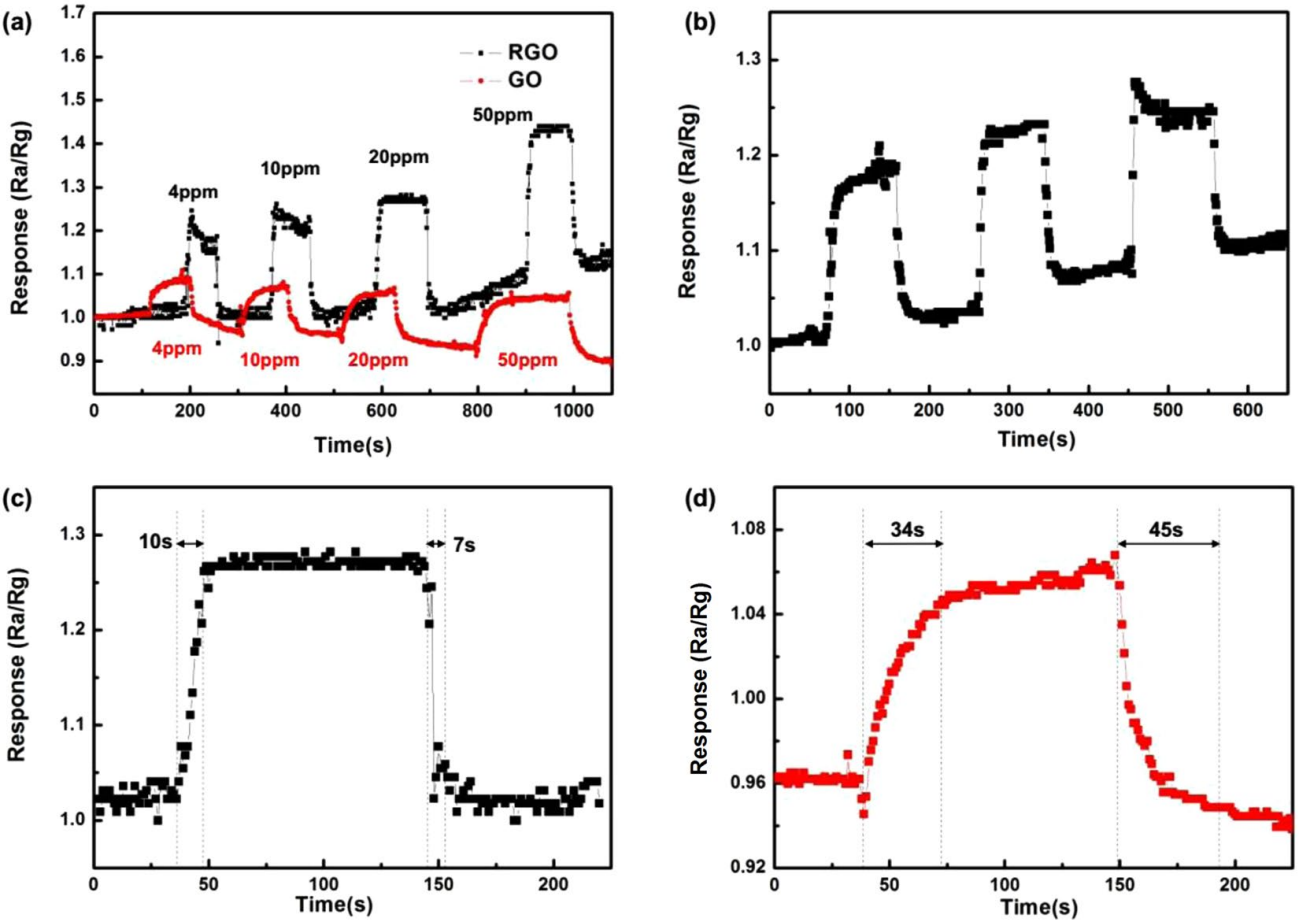
(**a**) Response and recovery curves of the sensor based on RGO and GO to various NO_2_ concentrations at room temperature. (**b**) The reproducibility of temporal response of RGO exposed to 4 ppm NO_2_ at room temperature. (**c**) and (**d**) The response recovery curves to 20 ppm NO_2_ of the sensor based on RGO and GO at room temperature, respectively.

**Figure 4:**
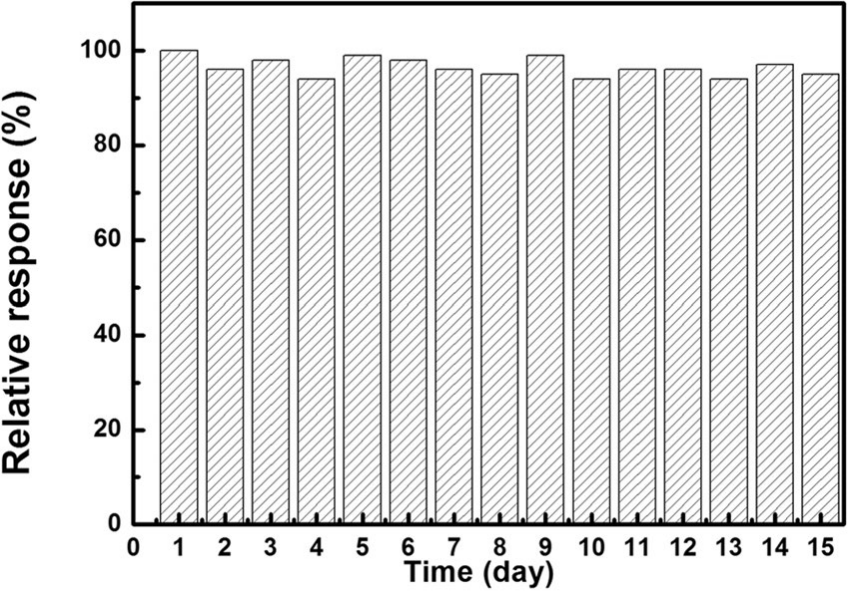
The long-time stability of RGO sensor for NO_2_ sensing.

**Figure 5:**
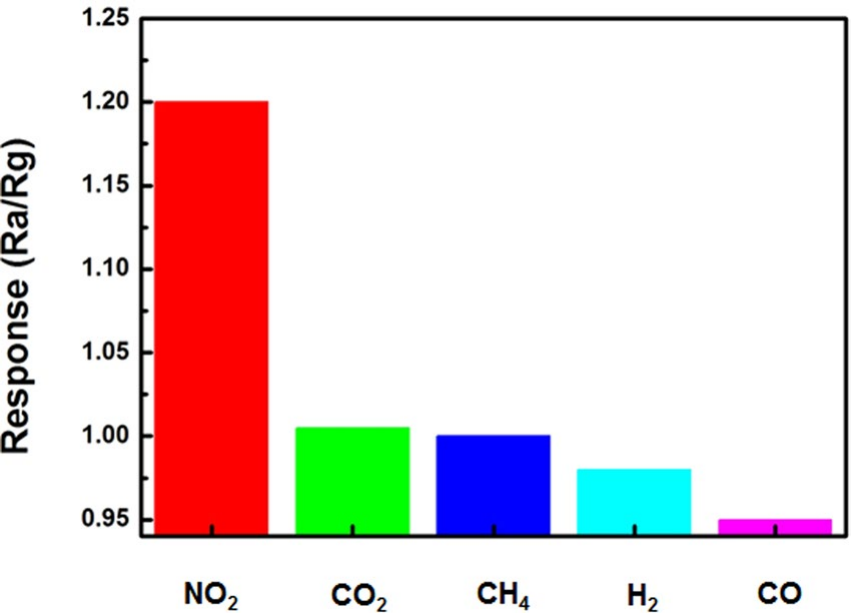
Detection selectivity of RGO sensor upon exposure to various vapors (~4 ppm) operating at room temprature.

Figure 3a shows the response and recovery curves to 4–50 ppm NO_2_ of the sensors at room temperature. It is seen that the response of the sensor based on RGO is larger than that of GO, indicating the enhancing sensing response by introduction of hierarchical nanostructures (The binding energy of GO and NO_2_ molecule is stronger than that of RGO, according to our calculations). The variety of response range is caused by two factors together, the increased specific surface area and the reduced oxygen containing groups. We can speculate that in the case of functional groups are invariant, the hierarchical nanostructures would improve the sensitivity of the device to a greater extent. As shown in Fig. 3b, the response variability range is almost constant for 3 cycles to 4 ppm NO_2_, indicating the reliability of our RGO NO_2_ sensor. Figure 3c,d shows the response and recovery curves to 20 ppm NO_2_ of the sensor based on GO and RGO hierarchical nanostructures at room temperature. The resistances of the devices were decreased upon exposure to NO_2_ gas, indicating NO_2_ doped the RGO film with holes, as RGO film exhibited the electrical behavior of a p-type semiconductor^1,28^. It is seen that the response of the sensor based on RGO hierarchical nanostructures is 1.27, which is higher than that of GO (1.06), indicating the enhanced sensing response by laser reduction of GO nanostructures. The response time and recovery time of the sensor based on nanostructured RGO are 10 s and 7 s, respectively, which are much shorter than that of GO (34 s and 45 s). Figure 4. shows response of the RGO sensor to 4 ppm NO_2_ for 15 days. It is seen that the response of the sensor floats slightly. The observed results reveal that RGO sensor exhibits good stability. The selectivity of the RGO sensor towards NO_2_ is also examined, as shown in Fig. 5. It is seen that the response of the sensor to 4 ppm NO_2_ is 1.2, which is much larger than those of the sensor to 4 ppm other gases, such as CO_2_, CH_4_, H_2_ and CO, indicating that the NO_2_ sensor exhibits good selectivity and can be used for selective detection of NO_2_. The sensing performances of the sensor were also compared with the previously reported sensors based on micro-nano structured graphene materials, as shown in Table 1.

**Table 1:** Comparison of sensing performances of our NO_2_ sensor with other published gas sensors based on micro-nano structured graphene-based materials.

properties of sensing material	Preparation method	Tested gas (ppm)	Gas response	Operating temperature(°C)	Response time(τ_r_/s)	Recovery time (τ_d_/s)	Year of publication	Ref.
Aerographite	Chemical vapour deposition	CO_2_ (500) NH_3_ (100) H_2_ (10000)	3.83% 1.21% 31.84% (ΔR/R_0_)	RT RT RT	3.98 8.7 0.25	6.92 11.58 0.35	2016	^26^
3D graphene foam network	Chemical vapour deposition	NH_3_ (1000)	30% (ΔR/R_0_)	RT	~500	~800	2011	^21^
3D graphene/SnO_2_	Freeze drying	NO_2_ (50)	6% (ΔR/R_0_)	RT	190	224	2014	^24^
3D SnO_2_/RGO	Freeze drying	NO_2_ (100)	1.079 (R_a_/R_g_)	55	>310^a^	373	2015	^25^
Graphene nanomesh	Nanosphere lithography	NO_2_ (10)	11%^a^ (ΔR/R_0_)	RT	>300^a^	>300^a^	2012	^22^
RGO nanofibers	Electrostatic self-assembly	NO_2_ (4.5)	20% (ΔR/R_0_)	RT	>300^a^	>300^a^	2014	^23^
Porous Graphene Oxide Network	Steam Etched	NO_2_ (250)	8% (ΔR/R_0_)	RT	~200^a^	~400^a^	2011	^20^
This work	Two-beam-laser Interference	NO_2_ (4) NO_2_ (20)	1.2 1.27 (R_a_/R_g_)	RT	<10 10	<10 7		

^a^Estimated from graphical plot.

As a comparison, RGO film reduced at 300 °C under the protection of nitrogen has similar oxygen content with that of RGO reduced by laser interference (shown in Figure S2). When expose thermal reduced RGO to 20 ppm NO_2_, the value of R_a_/R_g_ is 1.04, lower than that of reduced RGO by laser, which confirms the importance of nanostructures in the NO_2_ sensor. Based on the above results, the fabricated RGO sensor shows improved sensing response in NO_2_ and accelerated response/recovery rates. We also tested the response of gas sensors based on GO and RGO ethanol gas(Figure S3). Different from NO_2_, the resistances of the devices were increased upon exposure to ethanol gas, indicating ethanol doped the RGO film with electrons. As shown in Figure S4, binding energy of single layer graphene oxide decreased severely due to the shrinking of oxygen containing groups, sensitivity of RGO hierarchical nanostructures is lower than GO. The response and recovery times of the device fabricated by laser are improved obviously. Once again we confirmed that the hierarchical nanostructures could improve the response and recovery times of the device.

**Figure 6:**
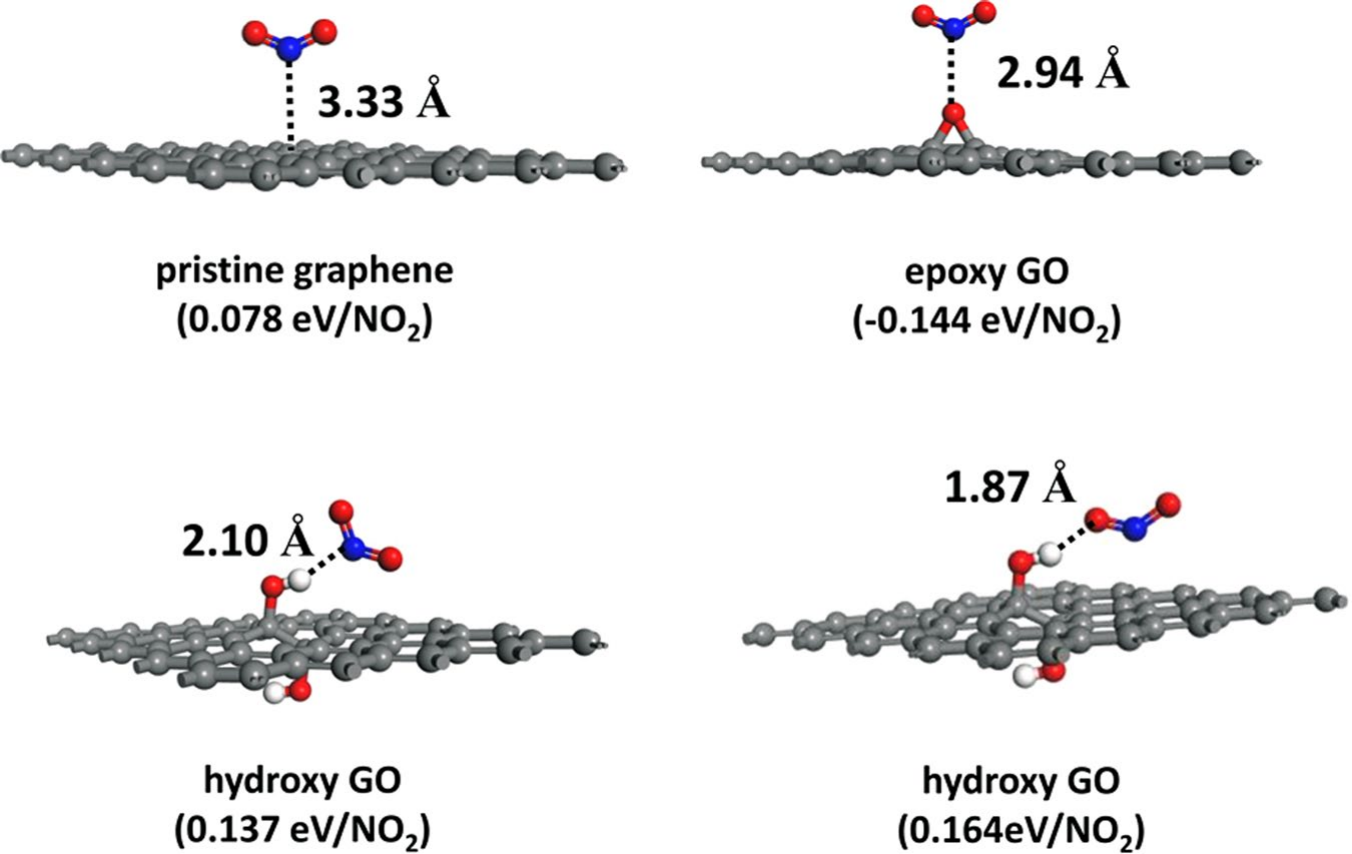
First-principle study of the interaction between NO_2_ molecule and graphene sheets, as well as epoxy or hydroxyl groups functionalized graphene.

To get further insight into the different mechanisms of the gas sensors, first principle study was carried out to give an essential explanation (Figure 6 and S4). In the present calculations. The binding energy between NO_2_ molecule and graphene is 78 meV, which is smaller than the interaction of NO_2_ and hydroxyl groups of graphene oxide. For epoxy group, a negative binding energy of −144 meV indicating a coulomb repulsion between epoxy and NO_2_ will push the molecules away from the GO sheet. Overall, according to other studies^29^, the oxygen functional groups on GO would hold significantly larger ability to absorb NO_2_ molecules than pure graphene. In this work, the RGO film shows a stronger response than GO film under the influence of the formation of nanostructure and the reduction of oxygen-containing functional groups. However, as shown in Figure S3, for ethanol, binding energy between a gas molecule and hydroxyl groups of graphene oxide is larger than the interaction of ethanol and graphene obviously, for the hydrogen bond between the oxygen group and hydroxyl of ethanol will decrease the energy.

## Conclusion

A graphene-based NO_2_ sensor has been successfully fabricated by TBLI reduction and nanostructuring of GO film. The presence of nanostructure increases the surface area and thus significantly improves the sensitivity of the device towards NO_2_, meanwhile, the response and recovery time is reduced to varying degrees at room temperature, compared to GO and thermal reduced RGO, this method was proved to be a novel approach to simultaneous reduction and nanostructuring of GO in a surfactant-free, mask-free and large-area manner. Our present study shows great potential for fabrication of high-performance room temperature graphene gas sensors.

## Methods

GO was produced via the Hummers method from natural graphite (Aldrich, <150 μm). The GO films were prepared by spin-coating GO solution on ceramic substrates with interdigital electrodes, at 1000 rpm for 30 s, dried at room temperature. The contacts are interdigital Ag-Pd electrodes as shown in the Figure S1(a). The minimum distance between contacts is about 0.2 mm. Then the sample was exposed by two beams which were split from the UV laser to reduce and produce nanostructures. A frequency-tripled, Q-switched, single-mode Nd:YAG laser (Spectra-physics) with about 10 ns pulse width was used for laser interference. 0.15 W laser power measured before the spectroscope. The exposure time was 10 s.

The dilute tested gas was air. Gas sensing properties were measured using a static test system. Saturated target vapor was injected into a test chamber (about 1 L in volume) by a microinjector through a rubber plug. After fully mixed with air (relative humidity was about 25%), the sensor was put into the test chamber. When the response reached a constant value, the sensor was taken out to recover in air. The electrical properties of the sensor were measured by CGS-8 intelligent test meter (Beijing Elite Tech. Co., Ltd, China) <250 mA. The response of a sensor was defined as the ratio (response: S = R_a_/R_g_) of the sensor resistance in air (R_a_) to that in the NO_2_ (or ethanol) gas (R_g_). The time taken by the sensor to achieve 90% of the total resistance change was defined as the response time in the case of adsorption and recovery time in the case of desorption. X-ray photoelectron spectroscopy (XPS) was performed using an ESCALAB 250 spectrometer. Scanning electron microscope (SEM) experiments were performed on a Hitachi S-4800 electron microscope. Optical microscope images were obtained from a Motic BE400 microscope. Raman spectra were measured with a Renishaw Raman microscope using 514 nm wavelength laser.

First principles calculations are based on generalized gradient approximation of Perdew-Burke-Emzerhof implemented in VASP code. Projector augmented wave method is used to describe the electron-ion interaction. The periodic graphene supercell (containing 50 carbon atoms) decorated with oxygen group is used to simulate the graphene oxide. In order to avoid the interaction with neighboring images, a vaccum layer of 20 Å is used. Cutoff energy with 500 eV for plane wave expansion and 2 × 2 × 1 Monkhorst-Pack mesh grid for Brillouin zone sampling are carried out for the calculations.

## Electronic supplementary material

Supporting Information
