# Supplementary material for: Improved NO2 Gas Sensing Properties of Graphene Oxide Reduced by Two-beam-laser Interference
Source: Sci Rep. 2018 Mar 20;8:4918. doi: 10.1038/s41598-018-23091-1 (PMC5861053; doi:10.1038/s41598-018-23091-1)
Supplement: Supplementary file 1 — Supporting Information [file 41598_2018_23091_MOESM1_ESM.doc]

**Supporting Information**

**Improved NO_2_ Gas Sensing Properties of Graphene Oxide Reduced by Two-beam-laser Interference**

Li Guo^1^, Ya-Wei Hao^1^, Pei-Long Li^1^, Jiang-Feng Song^1^, Rui-Zhu Yang^1^, Xiu-Yan Fu^2^, Sheng-Yi Xie^3^, Jing Zhao^4^ and Yong-Lai Zhang^2^

^1^ Institute of Materials, China Academy of Engineering Physics, Mianyang, Sichuan 621900, People’s Republic of China.

^2^ State Key Laboratory on Integrated Optoelectronics, College of Electronic Science and Engineering, Jilin University 2699 Qianjin Street, Changchun, 130012, People’s Republic of China.

^3^ Center for High Pressure Science and Technology Advanced Research, Changchun 130012, People’s Republic of China.

^4^ College of Electrical and Electronic Engineering, Changchun University of Technology, Changchun 130012, People’s Republic of China.

Correspondence and requests for materials should be addressed to Y.W.H. ([haoyawei@caep.cn](mailto:haoyawei@caep.cn)) or Y.L.Z ([yonglaizhang@jlu.edu.cn](mailto:yonglaizhang@jlu.edu.cn))

| Li Guo | 10guoli@163.com |
| --- | --- |
| Ya-Wei Hao | haoyawei@caep.cn |
| Pei-Long Li | lipeilong2012@126.com |
| Jiang-Feng Song | 16928306@qq.com |
| Rui-Zhu Yang | yangruizhu@caep.cn |
| Xiu-Yan Fu | [1054544189@qq.com](mailto:1054544189@qq.com) |
| Sheng-Yi Xie | ayikongjian@126.com |
| Jing Zhao | zhaojing@ccut.edu.cn |
| Yong-Lai Zhang | yonglaizhang@jlu.edu.cn |


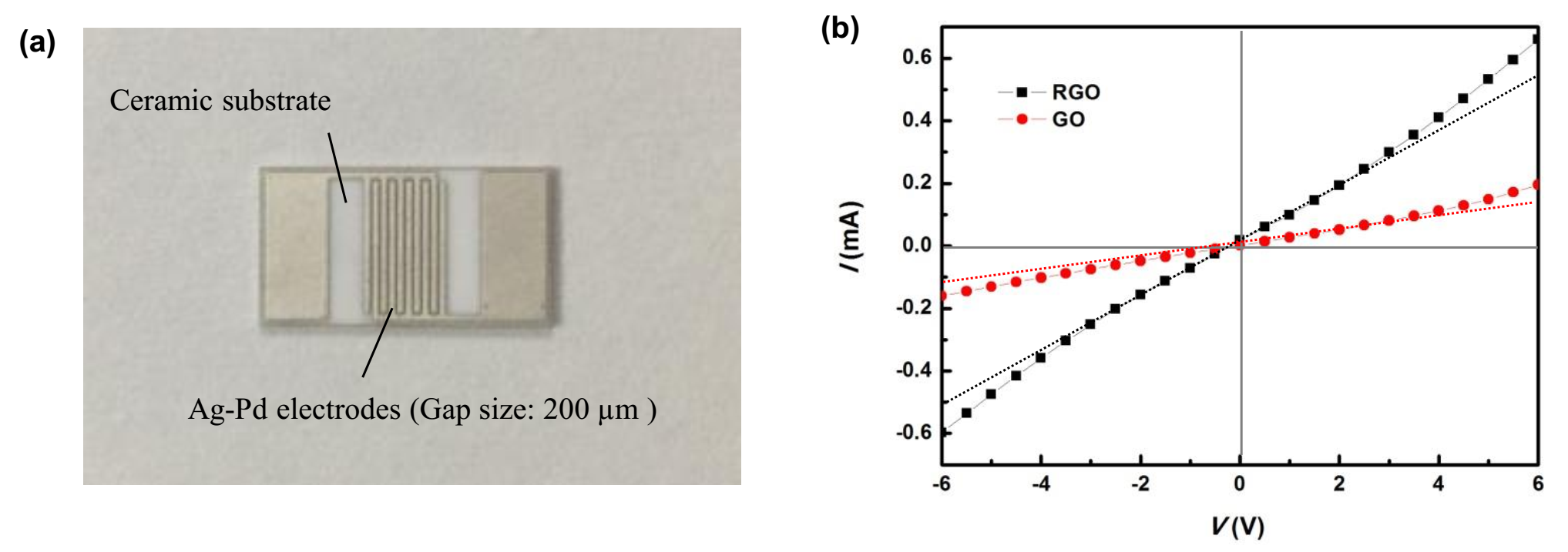


**Figure S1.** (a) Structure of [interdigital Ag-Pd electrode](http://www.baidu.com/link?url=4oV5oiSNBHbIQLPJ30VE5g7mdA4nbNwRtCmDXJHa120kYcst1yGE3YNwGYqL32IMUOTiCmzSjS5zTCxQPro8v_JP5XysSejr75vZdek5yA3sHgQRkKCeODyAjXHrpF4X)s. (b) I–V measurement of GO and RGO sensors.


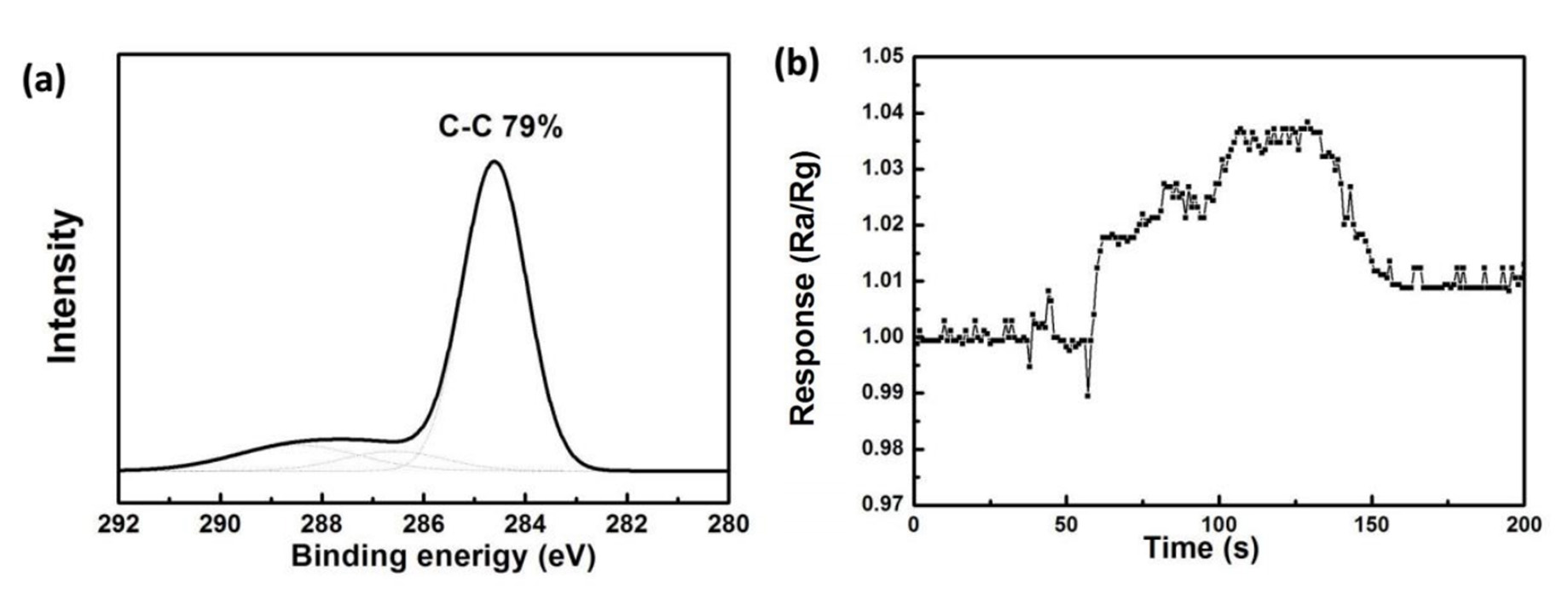


**Figure S2.** (a) C1s XPS spectrum of GO film reduced at 300 ºC under the protection of nitrogen. (b) The response recovery curves to 20 ppm NO_2_ of the sensor based on RGO without grating structure.


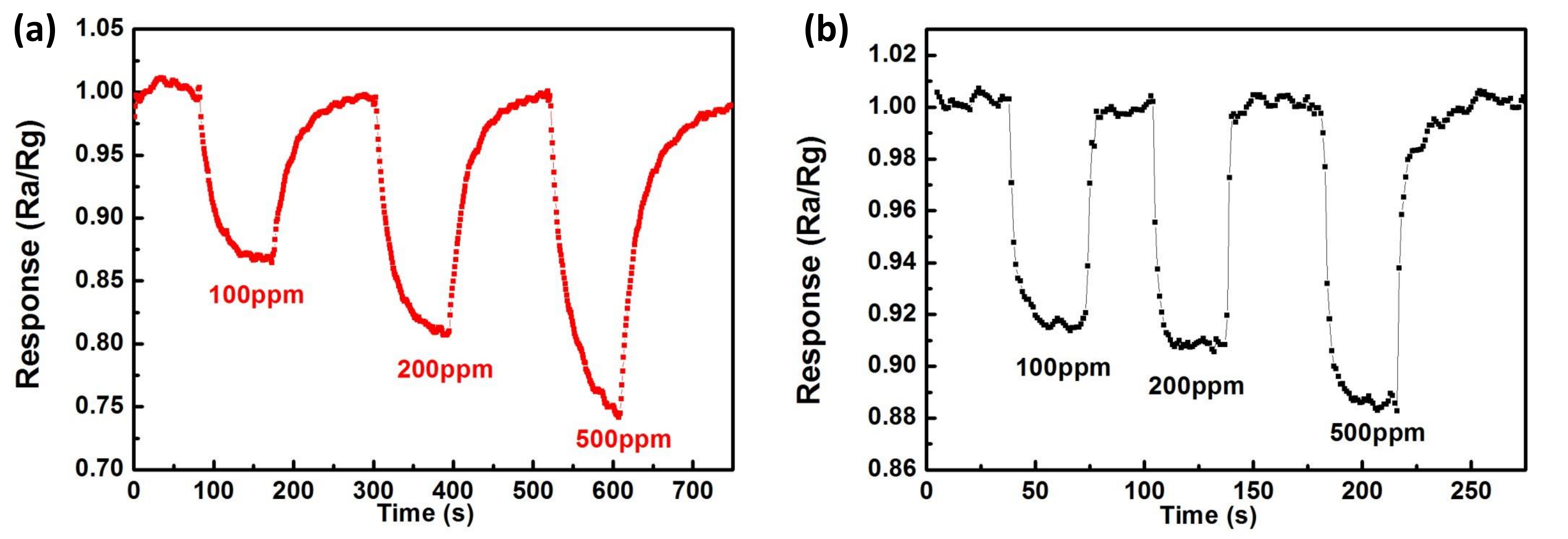


**Figure S3.** Response and recovery properties of the (a) GO and (b) RGO sensors to various ethanol concentrations gas at room temperature.


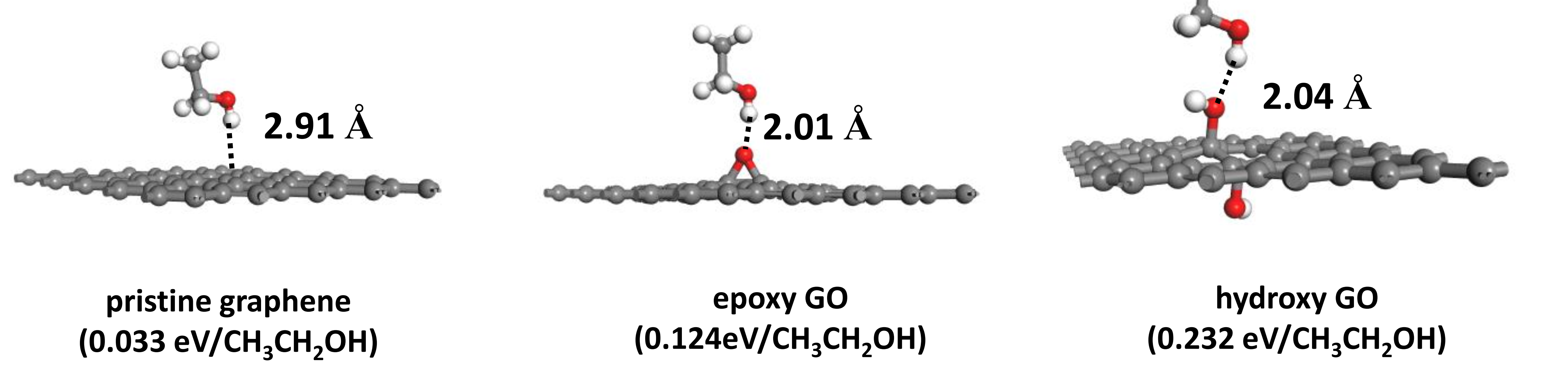


**Figure S4.** First-principle study of the interaction between ethanol molecule and graphene sheets, as well as epoxy or hydroxyl groups functionalized graphene.
